# Draft Genome Sequences of Two *Ralstonia pickettii* Strains with Different Aminoglycoside Resistance Phenotypes

**DOI:** 10.1128/genomeA.01257-16

**Published:** 2016-11-10

**Authors:** Ivone Vaz-Moreira, Javier Tamames, José Luis Martínez, Célia M. Manaia

**Affiliations:** a Universidade Católica Portuguesa, CBQF–Centro de Biotecnologia e Química Fina, Laboratório Associado, Escola Superior de Biotecnologia, Porto, Portugal; b LEPABE–Laboratório de Engenharia de Processos, Ambiente, Biotecnologia e Energia, Faculdade de Engenharia, Universidade do Porto, Porto, Portugal; c Centro Nacional de Biotecnología, Consejo Superior de Investigaciones Científicas (CSIC), Cantoblanco, Madrid, Spain

## Abstract

The genomes of two *Ralstonia pickettii* strains (H2Cu2 and H2Cu5), isolated from hospital effluent in a selective medium containing CuSO_4,_ were sequenced. They presented MICs of >256 and 6 µg/ml for the aminoglycoside gentamicin, respectively. The 5.2-Mb draft genomes have 40 contigs for strain H2Cu2 and 113 for H2Cu5.

## GENOME ANNOUNCEMENT

*Ralstonia pickettii* has been detected in water, plants, soils, and biological fluids (1–6). Infections caused by members of this species suggest their opportunistic character (6). Although still poorly studied, this species’ mechanisms for antibiotic resistance are supposedly mainly intrinsic. However, some resistance phenotypes, particularly those against aminoglycosides, vary within the species, suggesting that this resistance can be an acquired characteristic. This was a motivation to analyze the genomes of two isolates of *R. pickettii*, recovered from hospital effluent and yielding distinct aminoglycoside resistance phenotypes (1).

High-quality genomic DNA was extracted from purified cultures of *R. pickettii* strains H2Cu2 and H2Cu5 using the QIAamp DNA stool kit (Qiagen). After library construction and template preparation, the samples were sequenced with the Ion Torrent PGM system. Raw reads (2,725,428 for H2Cu2 and 2,865,510 for H2Cu5) were filtered according to their quality and trimmed by size (60 to 365 bp) using the software PRINSEQ (7). The high-quality trimmed reads (1,517,598 for H2Cu2 and 1,522,576 for H2Cu5) were assembled *de novo* using the Mira4 (8) and SPAdes version 3.6 (http://bioinf.spbau.ru/en/spades) assemblers. The contigs obtained with both assemblers were aligned and reordered with Mauve (http://darlinglab.org/mauve/mauve.html), using the complete genome sequence of strain *R. pickettii* 12J (NC_010682, NC_010683, NC_010678) as a reference. Considering the reference and the results of the SPAdes assembler, the contigs obtained with the Mira4 assembler were curated and edited with the Gap5 tool (Staden package version 1.2.14-r) (9), with a reduction in number and increase in length of some contigs. For strain H2Cu2, 40 contigs were obtained, resulting in a total genome size of 5.20 Mb, with 0.98 Mb being the largest contig size and with a total GC content of 64.0%. For strain H2Cu5, 113 contigs were obtained, resulting in a total genome size of 5.24 Mb , with 0.61 Mb being the largest contig size and with a total GC content of 63.7%. The completeness of each draft genome—99.29% for strain H2Cu2 and 99.97% for strain H2Cu5—was calculated with CheckM (10).

The similarity between both draft genomes was 91.81% according to the Average Nucleotide Identity (ANI) calculator (http://enve-omics.ce.gatech.edu/ani). Gene prediction and annotation were performed using the RAST server (http://rast.nmpdr.org/rast.cgi); 4,967 coding sequences for strain H2Cu2 and 5,144 coding sequences for strain H2Cu5 were identified. A function-based comparison revealed that the two strains share 92.8% of the genes. The aminoglycoside-resistant strain H2Cu2 presented genes associated with resistance to antibiotics, arsenic, and toxic compounds; genes related to lysozyme inhibitors; and genes related to phages/prophages. These genes could not be identified in strain H2Cu5. The presence of antimicrobial-resistance genes was also inferred based on a ResFinder 2.1 search (11) and a threshold value of 90%; the sequences showed significant identity with the beta-lactamase genes *bla*_OXA-22_ (94.5% for H2Cu2 and 95.2% for H2Cu5) and *bla*_OXA-60_ (90.8% for H2Cu2 and 100% for H2Cu5).

### Accession number(s).

The whole-genome shotgun projects for *Ralstonia pickettii* strains H2Cu2 and H2Cu5 were deposited in DDBJ/ENA/GenBank under the accession numbers MCGA00000000 and MCGB00000000, respectively. The versions described in this paper are the first versions, MCGA01000000 and MCGB01000000.

## References

[1] Becerra-Castro C, Machado RA, Vaz-Moreira I, Manaia CM 2015 Assessment of copper and zinc salts as selectors of antibiotic resistance in Gram-negative bacteria. Sci Total Environ 530–531:367–372. doi:10.1016/j.scitotenv.2015.05.102. 26057541

[2] Falcone-Dias MF, Vaz-Moreira I, Manaia CM 2012 Bottled mineral water as a potential source of antibiotic resistant bacteria. Water Res 46:3612–3622. doi:10.1016/j.watres.2012.04.007. 22534119

[3] Vaz-Moreira I, Nunes OC, Manaia CM 2014 Bacterial diversity and antibiotic resistance in water habitats: searching the links with the human microbiome. FEMS Microbiol Rev 38:761–778. doi:10.1111/1574-6976.12062. 24484530

[4] Coenye T, Vandamme P, LiPuma JJ 2002 Infection by *Ralstonia* species in cystic fibrosis patients: identification of *R. pickettii* and *R. mannitolilytica* by polymerase chain reaction. Emerg Infect Dis 8:692–696. doi:10.3201/eid0807.010472. 12095436 PMC2730328

[5] Stelzmueller I, Biebl M, Wiesmayr S, Eller M, Hoeller E, Fille M, Weiss G, Lass-Floerl C, Bonatti H 2006 *Ralstonia pickettii*–innocent bystander or a potential threat? Clin Microbiol Infect 12:99–101. doi:10.1111/j.1469-0691.2005.01309.x. 16441445

[6] Ryan MP, Pembroke JT, Adley CC 2006 *Ralstonia pickettii*: a persistent Gram-negative nosocomial infectious organism. J Hosp Infect 62:278–284. doi:10.1016/j.jhin.2005.08.015. 16337309

[7] Schmieder R, Edwards R 2011 Quality control and preprocessing of metagenomic datasets. Bioinformatics 27:863–864. doi:10.1093/bioinformatics/btr026. 21278185 PMC3051327

[8] Chevreux B, Wetter T, Suhai S 1999 Genome sequence assembly using trace signals and additional sequence information, p. 45–56. In Computer science and biology. Proceedings of the German Conference on Bioinformatics, GCB ’99. GCB, Hannover, Germany.

[9] Bonfield JK, Whitwham A 2010 Gap5—editing the billion fragment sequence assembly. Bioinformatics 26:1699–1703. doi:10.1093/bioinformatics/btq268. 20513662 PMC2894512

[10] Parks DH, Imelfort M, Skennerton CT, Hugenholtz P, Tyson GW 2015 CheckM: assessing the quality of microbial genomes recovered from isolates, single cells, and metagenomes. Genome Res 25:1043–1055. doi:10.1101/gr.186072.114. 25977477 PMC4484387

[11] Zankari E, Hasman H, Cosentino S, Vestergaard M, Rasmussen S, Lund O, Aarestrup FM, Larsen MV 2012 Identification of acquired antimicrobial resistance genes. J Antimicrob Chemother 67:2640–2644. doi:10.1093/jac/dks261. 22782487 PMC3468078

